# Biosensing Amplification by Hybridization Chain Reaction on Phase-Sensitive Surface Plasmon Resonance

**DOI:** 10.3390/bios11030075

**Published:** 2021-03-06

**Authors:** Ching-Hsu Yang, Tzu-Heng Wu, Chia-Chen Chang, Hui-Yun Lo, Hui-Wen Liu, Nien-Tsu Huang, Chii-Wann Lin

**Affiliations:** 1 Graduate Institute of Bioelectronics and Bioinformatics, National Taiwan University, Taipei 106, Taiwan; d02945009@ntu.edu.tw (C.-H.Y.); aresation@gmail.com (T.-H.W.); 2 Department of Medical Biotechnology and Laboratory Sciences, College of Medicine, Chang Gung University, Taoyuan 333, Taiwan; chang@mail.cgu.edu.tw; 3 Kidney Research Center, Department of Nephrology, Chang Gung Memorial Hospital, Taoyuan 333, Taiwan; 4 Department of Biomedical Engineering, National Taiwan University, Taipei 106, Taiwan; onlyby1231@gmail.com (H.-Y.L.); k8006780067@yahoo.com.tw (H.-W.L.); 5 Department of Electrical Engineering, National Taiwan University, Taipei 106, Taiwan; 6 Biomedical Technology and Device Research Laboratories, Industrial Technology Research Institute, Hsinchu 310, Taiwan

**Keywords:** aptamer, hybridization chain reaction, phase-sensitive surface plasmon resonance (pSPR) biosensor

## Abstract

Surface Plasmon Resonance (SPR) is widely used in biological and chemical sensing with fascinating properties. However, the application of SPR to detect trace targets is hampered by non-specific binding and poor signal. A variety of approaches for amplification have been explored to overcome this deficiency including DNA aptamers as versatile target detection tools. Hybridization chain reaction (HCR) is a high-efficiency enzyme-free DNA amplification method operated at room temperature, in which two stable species of DNA hairpins coexist in solution until the introduction of the initiator strand triggers a cascade of hybridization events. At an optimal salt condition, as the concentrations of H1 and H2 increased, the HCR signals were enhanced, leading to signal amplification reaching up to 6.5-fold of the detection measure at 30 min. This feature enables DNA to act as an amplifying transducer for biosensing applications to provide an enzyme-free alternative that can easily detect complex DNA sequences. Improvement of more diverse recognition events can be achieved by integrating HCR with a phase-sensitive SPR (pSPR)-tested aptamer stimulus. This work seeks to establish pSPR aptamer system for highly informative sensing by means of an amplification HCR. Thus, combining pSPR and HCR technologies provide an expandable platform for sensitive biosensing.

## 1. Introduction

The Surface Plasmon Resonance (SPR) biosensor is based on an electromagnetic wave that oscillates between the metallic film and the dielectric plate. The adsorption measurement on metal surfaces or metal nanoparticles (gold or silver) is based on SPR. Several color-dependent biosensor systems and different lab-on-chip sensors are key components. SPR is capable of performing real-time, label-free and high-sensitivity monitoring of molecular interactions and is now a leading technology for biomolecular target detection [1,2]. SPR has been employed for the detection of nucleic acids [3,4], proteins [5,6] and small molecules [4], by measuring the reflective index change during complex formation or dissociation [7,8]. However, the application of SPR to detect trace targets, particularly in complex biological samples, is hampered by non-specific binding and poor signal. A variety of approaches for amplification to support better detection have been explored to overcome this deficiency [6]. According to the operating principles, SPR biosensors can be divided into angle-, wavelength-, intensity- and phase-sensitive devices. With their complex optical configurations, pSPR sensors generally provide higher sensitivity and throughput, and have thus recently emerged as prominent biosensing devices [9]. pSPR is more sensitive than intensity-based SPR since the limit of detection (LOD) of pSPR is around 10^−7^ RIU, while the LOD of intensity-based SPR is at the 10^−5^ RIU scale [10,11]. In special designed and custom developed instrumentations, the SPR enables the detection of DNA targets down to the femtomolar range or lower [12,13].

Nucleic acid amplification methodologies have drawn significant research efforts for effective identification, as nucleic acids are regularly assessed to assist in biological experiments, molecular analysis and biomedical application. Therefore, the recognition of nucleic acids requires a powerful biosensor. A number of SPR studies were developed aiming for the incorporation of appropriate signal enhancement methods to determine complex conditions [14,15,16].

DNA aptamers are DNA sequences that bind to a certain protein screened by exponential enrichment systematic ligand evolution (SELEX) [17]. Aptamers have several advantages such as small size, versatile structure, strong binding affinity, high stability and good biocompatibility that has led to broad applications in the biomedical field [18]. Due to their simplicity and the ability to be easily modified and applied to different fields, aptamer-based SPR biosensors have been used to detect DNA [18], protein [19,20], nanoparticle [21], small molecules [22] and microRNA [23]. Compared with the conventional methods and other sensing strategies, this aptamer-based SPR biosensor system has several advantages such as faster, simpler, lower-cost, more practical, and better stability [24]. The proposed aptamer-based SPR biosensor could detect HIV-related DNA sensitively and specifically with a wide linear range from 1 pM to 150 nM and a detection limit of 48 fM, which is promising for the point-of-care detection of HIV infections [25]. Due to their simplicity and the ability to be easily modified and applied to different fields, the use of aptamer-based SPR biosensors can be a sensitive technique to improve the accuracy and correctness of measurements. However, the amplitude of signal detection is often unsatisfactory and requires continuous improvement to better meet the need for clinical applications [26].

Hybridization Chain Reaction (HCR) is a high-efficiency enzyme-free DNA amplification process operated at room temperature in which two stable species of DNA hairpins coexist in solution until a cascade of hybridization events is triggered by the introduction of the initiator strand [27]. Nonlinear HCR is considered to be a powerful signal amplifier for the detection of biomarkers through integration with flexible sensing platforms, given the benefits of its enzyme-free, high-order kinetic growth, high sensitivity, and simple procedure [28]. We introduce the methodology of HCR in which stable DNA monomers assemble only when exposed to a target DNA fragment.

The method of HCR has been applied to the detection of COVID-19. The technique is to provide a screening tool for isothermal amplification, a better screening strategy to prevent on-going epidemics. The chain reactions targeted Severe Acute Respiratory Syndrome coronavirus 2 (SARS-CoV-2) complementary DNA (cDNA) with loci corresponding to the gold standard of polymerase chain reaction (PCR) loci. The loop domain of the fuel hairpin molecules as optimization parameters, H1 and H2, the tunable segments in such responses, are used to enhance the hybridization efficiency. HCR reactions of the algorithm-derived with gel electrophoresis were validated. All proposed reactions show a hybridization complex with >1.5 k base pair molecular mass, which is clear evidence of a chain reaction. The trend shown by gel electrophoresis corresponds nicely to the algorithm’s simulated data. The HCR reactions and the corresponding algorithm form the basis for further sensing of SARS-CoV-2 [29]. Recently, usage of SPR in detection of SARS-CoV-2 has made significant progress [30,31,32,33]. However, the combination of SPR and HCR for the detection of SARS-CoV-2 has not been studied.

This paper describes a technique based on a chain reaction between three sets of DNA aptamer molecules for amplification, providing an enzyme-free alternative that can easily detect complex DNA sequences. HCR is performed to combine the platform of SPR signal detection as a simple implementable add-on technique that enables the increase in signal detection sensitivity. Improvement of more diverse recognition events can be achieved by integrating HCR with pSPR-tested aptamer stimuli. This feature enables DNA to act as an amplifying transducer for biosensing applications to establish a pSPR aptamer system for highly informative sensing by means of an amplification HCR.

## 2. Materials and Methods

### 2.1. Phase-Sensitive Surface Plasmon Resonance (pSPR)

The working principle is Heterodyne Interferometry, to obtain interference, a YVO4 birefringent crystal was adopted. Analysis of the related parameters affecting the efficiency of light coupling to the silicon waveguide’s Vertical-Cavity Surface-Emitting Laser (VCSEL, Philips ULM-852-BS-PL-S46FZP) with emission wavelength of 852 nm and a power of 2 mW is operated under a protection circuit with a 300 ohm resistance. This 852 nm light propagates along the vertical direction into the birefringent crystal, and p-polarization and s-polarization rays exhibit different optical path lengths (OPD) and subsequently shift in phase relative to one another. To accurately distinguish detection from the background signal, the thermal drift issue was subtracted by adding a reference arm, where light passes a beam splitter. The other part of the laser light goes to the fluidic system. Phase modulation was yielded by direct current modulation on VCSEL [9]. The residual amplitude modulation (RAM) issue was dealt with using the Generalized Lock-In Amplifier algorithm (GLIA), a method previously published by our group to establish a band-pass filter in GLIA to exclude out-of-band noise and to improve the signal-to-noise ratio [34]. The SPR sensor chip is composed of a 49 nm gold plasmonic layer and a 2 nm chromium adhesion layer on BK-7 glass. The thickness of the gold layer is chosen to optimize the sensitivity of the phase detection.

**Figure 1:**
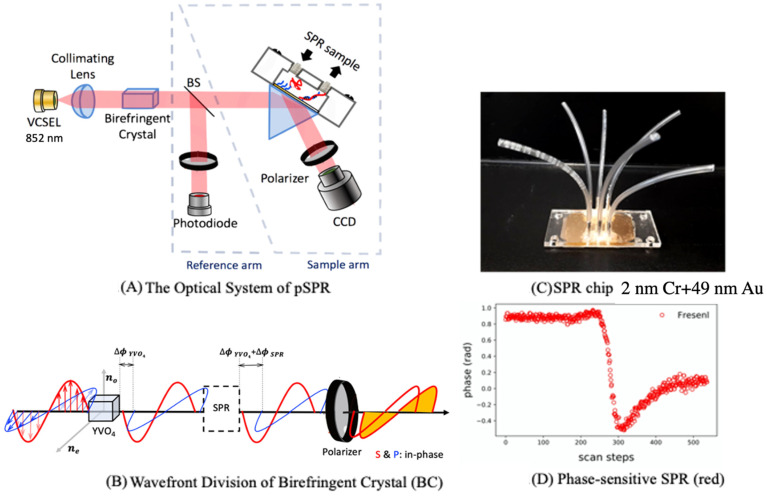
(**A**) Setup of the phase-sensitive surface plasmon resonance (pSPR) system. (**B**) Wavefront division of the birefringent crystal. (**C**) SPR chip. (**D**) Phase-sensitive SPR scan steps.

The gold films were deposited by an electron beam evaporator at a vacuum level of approximately 3 × 10^−6^ Torr [35]. The deposition was 0.2 Å/s. To elucidate the sensitivity of the SPR sensor chips, angle-resolved phase spectra were measured and fitted with Fresnel’s multilayer reflection model (Figure 1). The efficacy of the system was built first to estimate the wavelength-to-current sensitivity factor (S) value, which is intrinsic to the nature of individual VCSEL; we determined a specific current modulation that is needed to achieve Δ′′ = 3.8317. The feasibility of the pSPR system through fringe image and SPR image was at the Krestchmann angle. To accurately distinguish the DNA measurement signal, thermal drift was subtracted by adding a reference arm. Then, with glucose solution for sensitivity calibration, a Limit-of Detection (LOD) of 7.5 × 10^−7^ RIU was demonstrated where noise was estimated to be around 0.0004 rad. Using this system, a detection of target DNA down to 50 nM can be reached.

In order to obtain the interference of light, a birefringent crystal (BC) was used. The laser light propagates along the vertical direction into BC, and p-polarization and s-polarization rays exhibit different optical path lengths (OPD) and are subsequently shifted in phase relative to one another. To accurately distinguish the detection signal from the background signal, the thermal drift was subtracted by adding a reference arm, where light passes a beam splitter (BS). The other part of the laser light goes to the fluidic chip, where pSPR measurements could be done.

### 2.2. DNA Aptamers

The DNA aptamer oligonucleotides were synthesized and purified by HPLC at Purigo Biotech, Inc. (Taipei, Taiwan), and their sequences are listed in Table 1. A stock solution of the thiol-modified hairpin probe was prepared in a 10 mM Tris-HCl buffer solution (pH 8) containing 1 M NaCl and 0.1 M MgCl_2_. These chemicals were obtained from Sigma-Aldrich (St. Louis, MO, USA). All DNA stock solutions were stored at 4 °C to prevent degradation.

**Figure 2:**
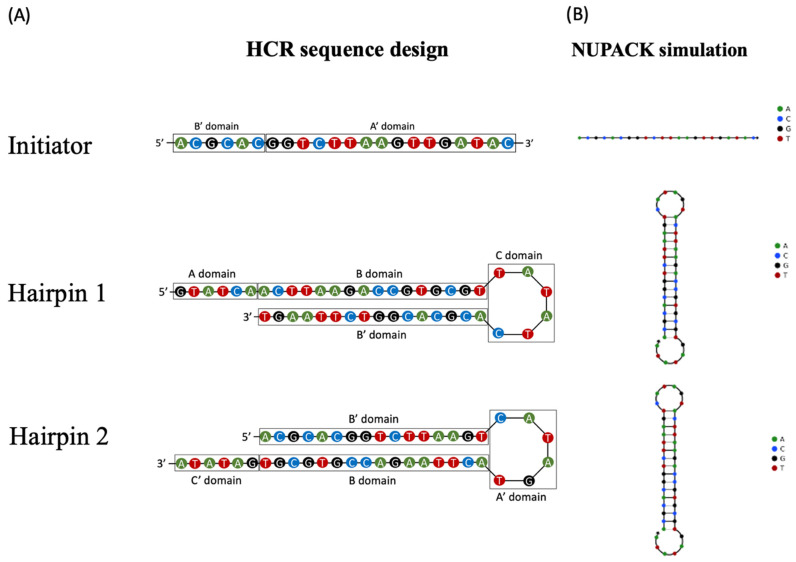
The design of three DNA aptamers that exhibit HCR signal amplification capabilities. (**A**) Schematic illustration of the HCR-pSPR detection aptamers. (**B**) Simulative schematics of the bare aptamer and the coupling DNA modified aptamer.

The folding of the DNA initiator and two hairpins was predicted using Nupack (available at http://www.nupack.org/, accessed on 9 October 2020) (Figure 2) [17]. We used cancer metastasis exosome Integrin α6β4 biomarker to validate and implement the HCR-based SPR-Biosensor and as the positive control for the experiment [36,37].

### 2.3. HCR on pSPR with Pre-Immobilized Initiator Probe

The procedure of probe immobilization and target hybridization was done first to anneal a gold film surface with deionized water to avoid non-specific binding and 20 mM 6-mercapto-1-hexanol (MCH) (Sigma-Aldrich, St. Louis, MO, USA) to minimize generation of a secondary structure. Then, 1 uM initiator probe DNA was added for 1 h for a higher density of immobilization. Again, the gold film surface was cleaned with deionized water to further reduce non-specific binding. With the probe immobilized, DNA hybridization can then be measured by the pSPR system.

**Figure 3:**
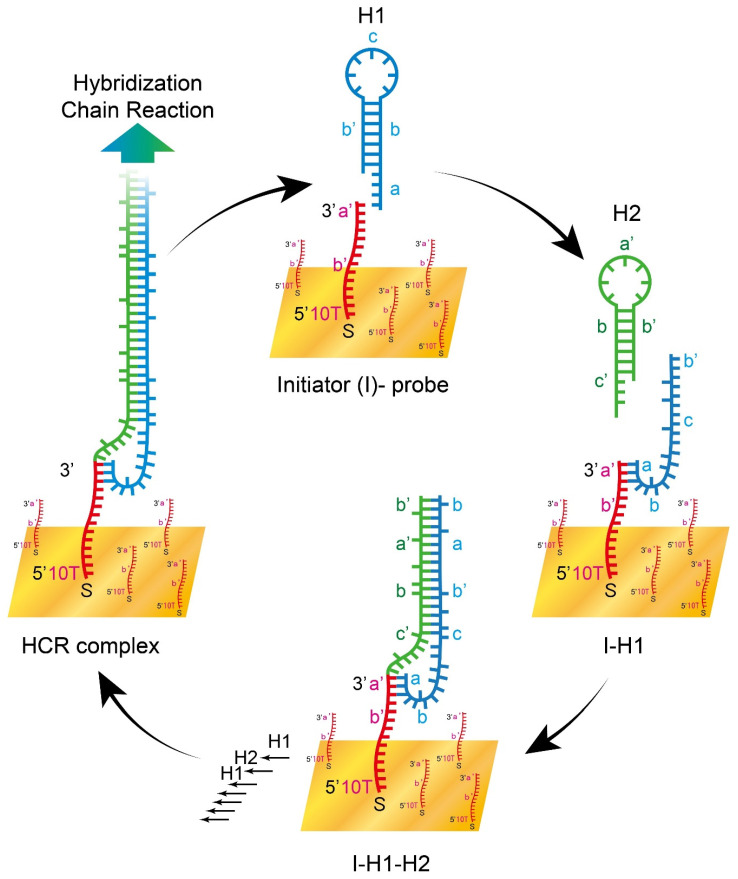
Schematic illustration of the HCR-pSPR self-assembly on gold surface detection platform.

We designed HCR sequences and anchored 1 μM 5′-end thiolated-initiator and 10 nM thiolated-10 thymine on the gold surface of the pSPR chip, and then introduced 1 μM of H1. Later, a mixture of 1 μM H1 and H2 was injected to enable HCR reaction. As the initiator was bound by H1, forming an initiator-H1 complex, H2 was then combined to the H1 tail, and H1 and H2 were bounded alternatively and repeatedly to form a double helix DNA complex structure, leading to signal amplification (Figure 3). A gradient of sodium chloride was injected into the fluid system as a calibration ruler for data normalization. After the definition of a working angle, signals of self-assembly were captured by a Charge-coupled Device camera and recorded for analysis.

### 2.4. DNA Gel Electrophoresis

To verify the initiator, H1 and H2 DNA aptamers underwent HCR amplification. The 2% agarose gel electrophoresis experiment was performed in 1× sodium borate buffer (1× SB buffer) (Sigma-Aldrich, St. Louis, MO, USA). Electrophoresis was done with a 100 V driving voltage (with MAJOR SCIENCE MP-100) at room temperature, using a 1× SB running buffer. The fluorescent dye used for imaging was Safe Green (Hycell, Taipei, Taiwan). The proportion of initiator, H1 and H2 was 5:5:0.25 to 5:5:10, and after 30 min of mixing, the HCR yield was from 100 bp to 2 kbp. In the gel electrophoresis experiment, the HCR chemistry was in free solution, the proportion of initiator, H1 and H2 was 5:5:0.25 to 5:5:10, while in pSPR, the initiator was anchored on a gold surface, a two-dimensional space with 10 mer Thymine spacers in between.

## 3. Results

### 3.1. Validation of HCR on pSPR with Pre-Immobilized Initiator Probe

**Figure 4:**
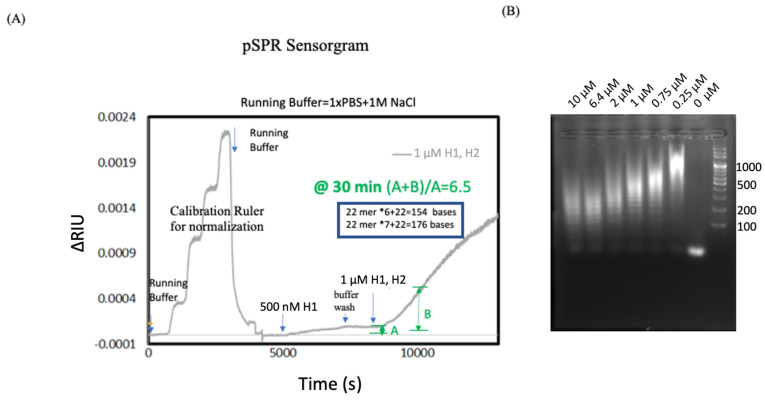
(**A**) Real-time detection of the HCR reaction. In the pSPR result, after 30 min of the initiator and H1 binding reaction and the 30 min washing step marks A, the time point of 30 min after the addition of the H1 and H2 mixture marks B. (A + B)/A = 6.5, indicating a 6.5-fold of the detection measure at 30 min. (**B**) Combining pSPR and DNA gel electrophoresis results. The length of the HCR complex at the same reaction time, 30 min, can be observed.

We anchored the HCR initiator DNA aptamer on the SPR chip gold surface and added H1 and H2 sequences to amplify the HCR signal. A gradient of sodium chloride, 0.025 M NaCl, 0.05 M NaCl, 0.1 M NaCl and 0.2 M NaCl was injected into the fluidic system to become a calibration ruler for data normalization. Running buffer (1 × PBS + 1 MNaCl) was added to gain the baseline. At around 5000 s, 500 nM H1 was injected to form the H1-initiator complex. After one hour, running buffer was added to stop the reaction; the reaction was around 0.0001 ∆RIU. We then added 1 µM mixture of H1 and H2 to the system. As can be seen, the HCR reaction was around 0.0005 ∆RIU at 30 min. In the pSPR result, after 30 min of the initiator and H1 binding reaction and the 30 min washing step marks A, the time point of 30 min after the addition of H1 and H2 mixture marks B. (A + B)/A = 6.5, indicating a 6.5-fold of the detection measure at 30 min. There was no significant signal difference when only H1 was added (Figure 4A).

### 3.2. Combining pSPR and DNA Gel Electrophoresis

By combining pSPR and DNA gel electrophoresis results, we were able to study the length of HCR product at the same reaction time of 30 min. We also compared the product length of HCR at 60 min, by pSPR and DNA gel electrophoresis. Initiator was 22 + 10 T = 32 mer, but only 22 mer reacted in the HCR process. H1 and H2 each had 44 mer, the combination of H1 and H2 makes 22 + 22 = 44, 22 × 6 + 22 = 154 bases, 22 × 7 + 22 = 176 bases (Figure 4B).

### 3.3. The Effect of Aptamer and Salt Concentration on HCR Amplification

**Figure 5:**
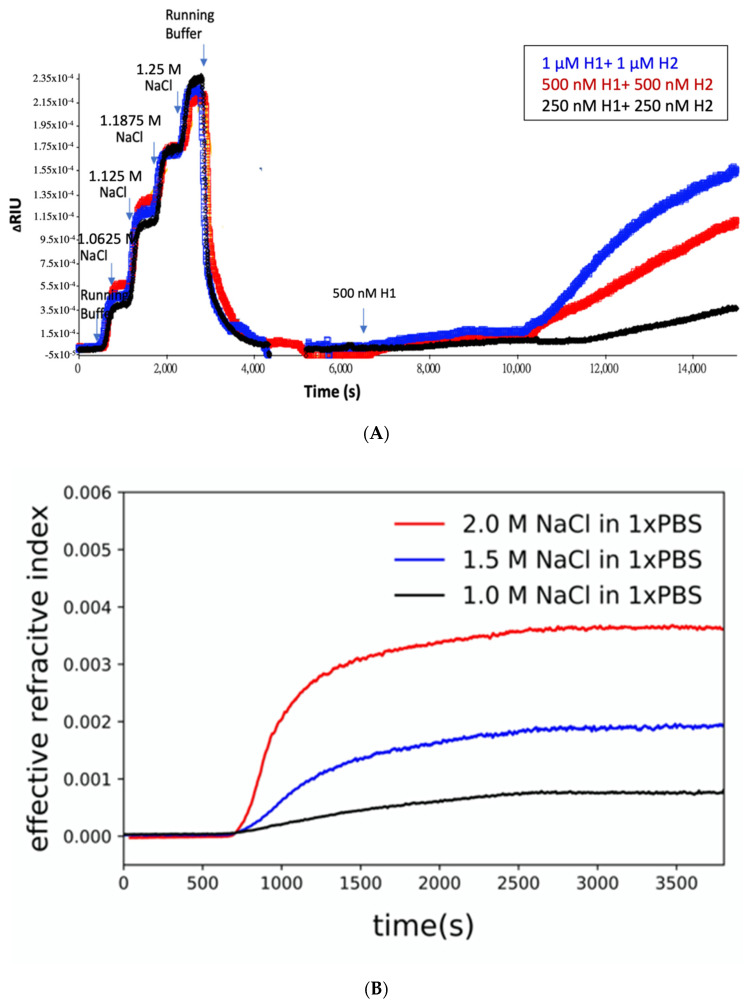
Result of HCR reaction under different aptamer concentrations and ionic strength environments. (**A**) The concentration of H1, H2 and the subsequent HCR amplification are in direct proportion: while the concentration of H1 and H2 increases, HCR amplification increases. Blue: 1 µM H1+ 1 µM H2, Red: 500 nM H1 + 500 nM H2 and Black: 250 nM H1 + 250 nM H2. (**B**) Validation of salt concentration of HCR on pSPR with pre-immobilized initiator probe; 1.0 M, 1.5 M and 2.0 M NaCl in 1× PBS were added with 1 µM H1 and H2 mixture at 400 s, and injected 1× PBS buffer at 2530 s. Three HCR reactions showed 0.0007, 0.0018 and 0.0036 in Δ RIU indicating different salt concentrations affected HCR amplification results.

Results of HCR reaction under different aptamer concentrations and ionic strength environment are shown in Figure 5. The results showed that the concentrations of H1, H2 and the subsequent HCR amplification were in direct proportion: while the concentrations of H1 and H2 increased, HCR amplification increased (Figure 5A). In this set of experiments, the initiator probe was fixed on the SPR chip gold surface. The chip was then set for sensing under changing salt concentrations. As shown in Figure 5, the baseline was established using a running buffer. After the baseline was established, H1 + H2 mixture samples were then injected into the microfluidic channel for sensing. At around 700 s, H1 + H2 mixture samples arrived. Upon sample arrival, pSPR rising signals were observed corresponding to different experimental conditions. In order to validate the effect of salt concentration, HCR was undertaken under 1.0, 1.5 and 2.0 M NaCl in 1× PBS. As can be seen, the HCR reaction was most intense under a running buffer composition of 1.5 M NaCl in 1× PBS. Based on the data, the HCR reaction was relatively smaller under salt concentrations higher or lower than 1.5 M NaCl in 1× PBS (Figure 5B). Therefore, we concluded that there is an optimized salt concentration for surface-based HCR.

## 4. Discussion

In the present work, a pSPR detection system based on HCR was developed for the amplification of DNA aptamers. We designed HCR sequences and anchored the 5′-end thiolated-10 thymine-initiator on the gold surface of the pSPR chip, and then we introduced H1 and H2 hairpin DNA aptamers to enable HCR reaction. As the initiator was bound by H1, forming an initiator-H1 complex, H2 was then combined to the H1 tail, and the H1 and H2 were bounded alternatively and repeatedly to form a double-helix DNA complex structure without an enzyme. Finally, as the concentration of H1 and H2 increased, the HCR signals were enhanced, leading to signal amplification.

The HCR chemistry was in free solution in the gel electrophoresis experiment. The proportion of initiator, H1 and H2 was 5:5:0.25 to 5:5:10, while the initiator was anchored on the gold surface in pSPR, a two-dimensional space with 10 mer adenosine spacers in between.

We designed HCR sequences, planted thiolated-initiator on the gold surface of the pSPR chip, and then introduced H1 and H2 to enable HCR. On the other hand, when thiolated H1 was first bound to the gold surface, HCR could not be activated followed by adding initiator and H2 in our setting. This is because the initiator is needed initially when the hairpin structure is unlocked, and the structure of HCR-initiator can be revealed and opened for the HCR reaction.

The HCR response was most intense under a running buffer composition of 1.5 M NaCl in 1× PBS, as can be seen. Based on the data, at salt concentrations greater than or less than 1.5 M NaCl in 1× PBS, the HCR reaction was relatively smaller. We therefore conclude that, for pSPR-based HCR, there is an optimal salt concentration.

Loop-mediated isothermal amplification (LAMP) has also observed these phenomena [38,39,40,41,42]. This effect was the result, according to the authors, of an optimal salt concentration. Therefore, the salt concentration must be optimized to achieve maximum sensitivity when applying HCR reactions for diagnostic purposes. Recent studies have demonstrated the usefulness of the sequence specimen during genetic detection by combining LAMP with one stage stream displacement (OSD) [43]. However, because OSD is unable to provide signal amplification, the signal-to-noise ratio or the observed signals may not be significant as to satisfy practical use. In order to meet this challenge, a more advanced sensing principle has been developed to replace OSD with HCR. The highly contagious norovirus (NoV) was used as a model target to achieve more reliable detection. Compared to LAMP-OSD, LAMP-HCR can detect as few as 30 copies of the NoV gene in fecal samples with significantly increased signal amplification and a signal-to-background ratio [43]. Furthermore, because of the high compatibility of HCR, the final LAMP-HCR products can be flexibly transduced into different types of readings. In the future, combination techniques of pSPR and LAMP-HCR can be further evaluated in order to expand the detection to a higher tolerance for complicated biological materials.

In a previous study using microfluidics, higher SPR signals could be obtained with target concentrations from 10 nM to 100 nM where target signals can be detectable prior to HCR [12]. During HCR in the SPR microfluidics, a relatively quick steady-state can be reached. Signal flattening is reached at concentrations higher than 100 nM due to surface saturation enabled by the efficient SPR microfluidics used for target hybridization. These data demonstrate that performing HCR does decrease the detection limit for the bound target. Thus, when using higher-performance SPR equipment and microfluidics HCR combination [13], it is reasonable to expect that the limit of detection could reach far below the nanomolar level.

A recent advance of pSPR has been proposed to combine amplification cascades of catalyzed hairpin assembly (CHA) and HCR with the sensitive SPR responses of plasmonic gold nanorods [23]. The proposed bioassay exhibited ultrahigh sensitivity toward tested miRNA with dynamic range from 5.0 × 10^−17^ M to 1.0 × 10^−11^ M.

Thus, the method of combining pSPR and HCR technologies provides an expandable platform for future direction of ultrasensitive biosensing.

## 5. Conclusions

HCR is a high-efficiency enzyme-free DNA amplification process operated at room temperature, in which two stable species of DNA hairpins coexist in solution before the introduction of the initiator strand causes a cascade of hybridization events. Improvement of more diverse recognition events can be achieved by integrating HCR with pSPR-tested aptamer stimuli. This feature enables DNA to act as an amplifying transducer for biosensing applications. This work sets out a pSPR system for highly informative sensing using HCR for amplification and opens a new era of sensitive detection for various biological and medical conditions for pSPR biosensor technology. Possible future applications may include early diagnosis of cancer metastases, prognosis prediction and exosome profiling by tumor marker quantification.

## Figures and Tables

**Table 1:** Sequence of the DNA aptamers used in this research.

Aptamer Sequence (5′→3′)	
Initiator	
(thiolated-10T + 22 mer)	HS-TTTTTTTTTTACGCACGGTCTTAAGTTGATAC
H1 (44 mer)	GTATCAACTTAAGACCGTGCGTTATATCACGCACGGTCTTAAGT
H2 (44 mer)	ACGCACGGTCTTAAGTTGATACACTTAAGACCGTGCGTGATATA

## Data Availability

Not applicable.
